# Acute shock caused by *Clonorchis sinensis* infection: a case report

**DOI:** 10.1186/s12879-019-4644-5

**Published:** 2019-11-29

**Authors:** Nan Wang, Bin Tang, Yuhua Hao, Xue Bai, Xuelin Wang, Yuxiang Li, Yong Yang, Shicun Li, Shuo Hao, Xinyu Wang, Mingyuan Liu, Xiaolei Liu

**Affiliations:** 1 Key Laboratory of Zoonosis Research, Ministry of Education, Institute of Zoonosis/College of Veterinary Medicine, Jilin University, Changchun, 130000 China; 2 Affiliated Zhongshan Hospital of Dalian University, Dalian, 116000 China; 3 Infection Department, the First Hospital of Jilin University, Changchun, 130021 China; 4 Jiangsu Co-innovation Center for Prevention and Control of Important Animal Infectious Diseases and Zoonoses, Yangzhou, China

**Keywords:** Clonorchiasis, Cholecystitis, Acute shock, PCR

## Abstract

**Background:**

Clonorchiasis, caused by *Clonorchis sinensis* (*C. sinensis*) infection, is a serious food-borne zoonotic disease that is often asymptomatic or shows only mild symptoms, which leads to delayed treatment and chronic clonorchiasis and results in various complications, such as cholelithiasis, cholangitis, cholecystitis and cholangiocarcinoma. However, acute shock caused by *C. sinensis* infection has not been reported. Here, for the first time, we describe a fatal case of acute shock caused by *C. sinensis* infection.

**Case presentation:**

A patient with a history of eating raw or undercooked freshwater fish was hospitalized with acute shock caused by severe abdominal pain. Physical examination suggested acute abdomen with severe abdominal pain and rigidity. Computed tomography (CT) detection indicated acute cholecystitis and cholelithiasis. After cholecystectomy, several liver flukes were found in the drainage tube. Furthermore, morphological analysis and polymerase chain reaction (PCR) identified the pathogen as *C. sinensis*. The liver gradually restored normal function after anthelmintic therapy with praziquantel.

**Conclusions:**

In this fatal case, *C. sinensis* infection was the cause of acute shock, which is rarely found in the clinic environment. This report aims to increase awareness of the hazards and complications related to clonorchiasis. The PCR diagnosis method used in this report might be helpful in reducing the misdiagnosis of clonorchiasis and unnecessary cholecystectomy.

## Background

Clonorchiasis is a serious food-borne parasitic disease caused by *C. sinensis* infection, and humans become infected by *C. sinensis* after ingesting raw or semi-raw freshwater fish and shrimp that contain infective metacercariae [1]. In 2009, the International Agency for Research on Cancer classified *C. sinensis* as group I carcinogen [2]. Approximately 15 million people are infected with *C. sinensis* worldwide, especially in Southeast Asia, with 13 million people being infected in China alone [3, 4]. Clonorchiasis is often asymptomatic or shows only mild symptoms, such as indigestion, nausea, diarrhea, and abdominal discomfort [5]. The absence of typical symptoms may delay the diagnosis and treatment of the disease and may eventually lead to chronic infection. Currently, the gold standard for diagnosing *C. sinensis* infection is detecting eggs in stool [6]. However, the likelihood of misdiagnosing *C. sinensis* is high due to the low sensitivity of the detection of eggs in stool, and misdiagnosis could lead to chronic infection. Chronic infection results in biliary obstruction, cholelithiasis, cholangitis and cholecystitis, which are risk factors for cholangiocarcinoma (CCA) [7, 8]. In the coming decades, nearly 5000 cases of CCA attributed to *C. sinensis* infection could occur in East Asia annually [9]. The obstruction of the biliary tract may lead to a bile and pancreatic juice countercurrent, which results in cholecystitis and pancreatitis [10, 11]. However, acute shock caused by *C. sinensis* infection has not been reported. In this report, we present a fatal case of acute shock caused by *C. sinensis* infection. The PCR diagnosis method used in this report might be helpful for the diagnosis of clonorchiasis.

## Case presentation

**Fig. 1:**
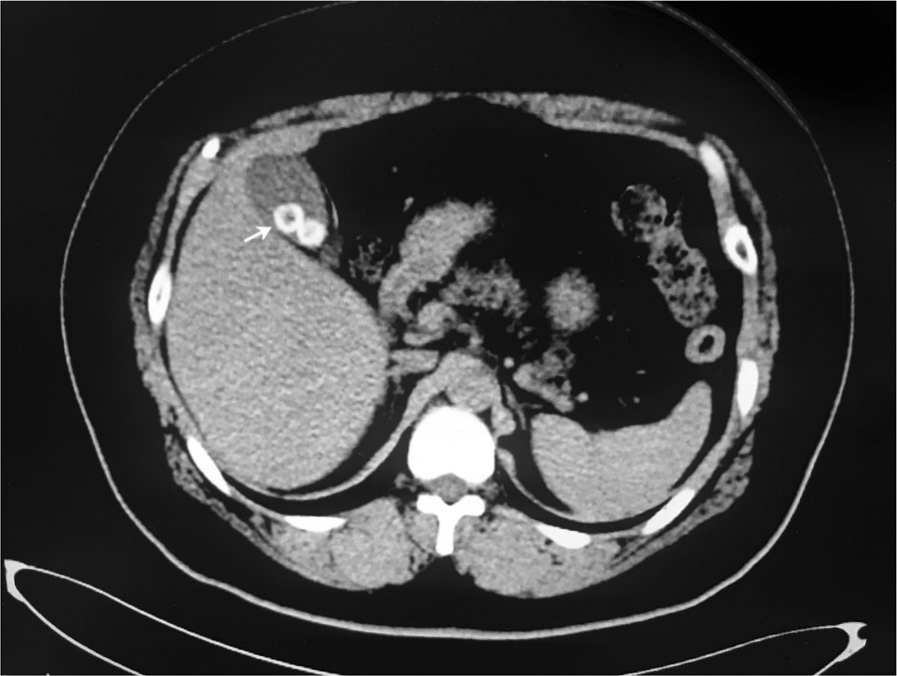
CT image showing stone-like substance in the gallbladder (white arrow)

**Fig. 2:**
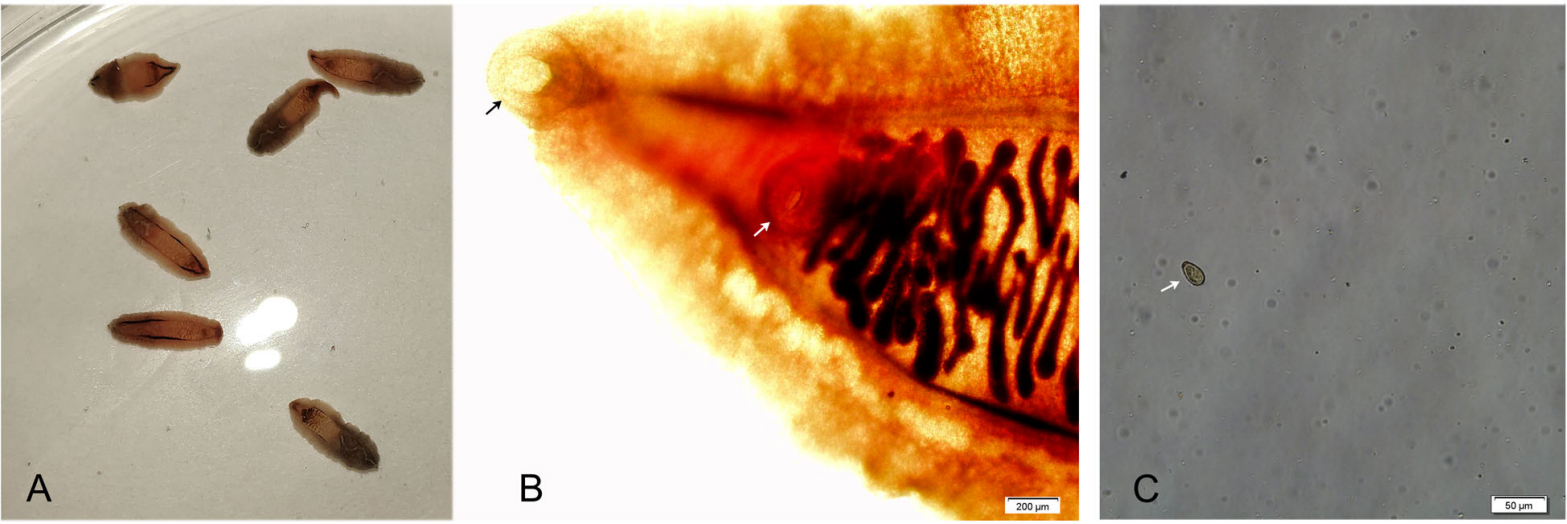
Morphologic observation of *C. sinensis* worms and egg. **a** *C. sinensis* worms drained from bile. **b** The morphologic features of worm with oral sucker (black arrow) and ventral sucker (white arrow). **c** Egg of *C. sinensis* from bile

**Fig. 3:**
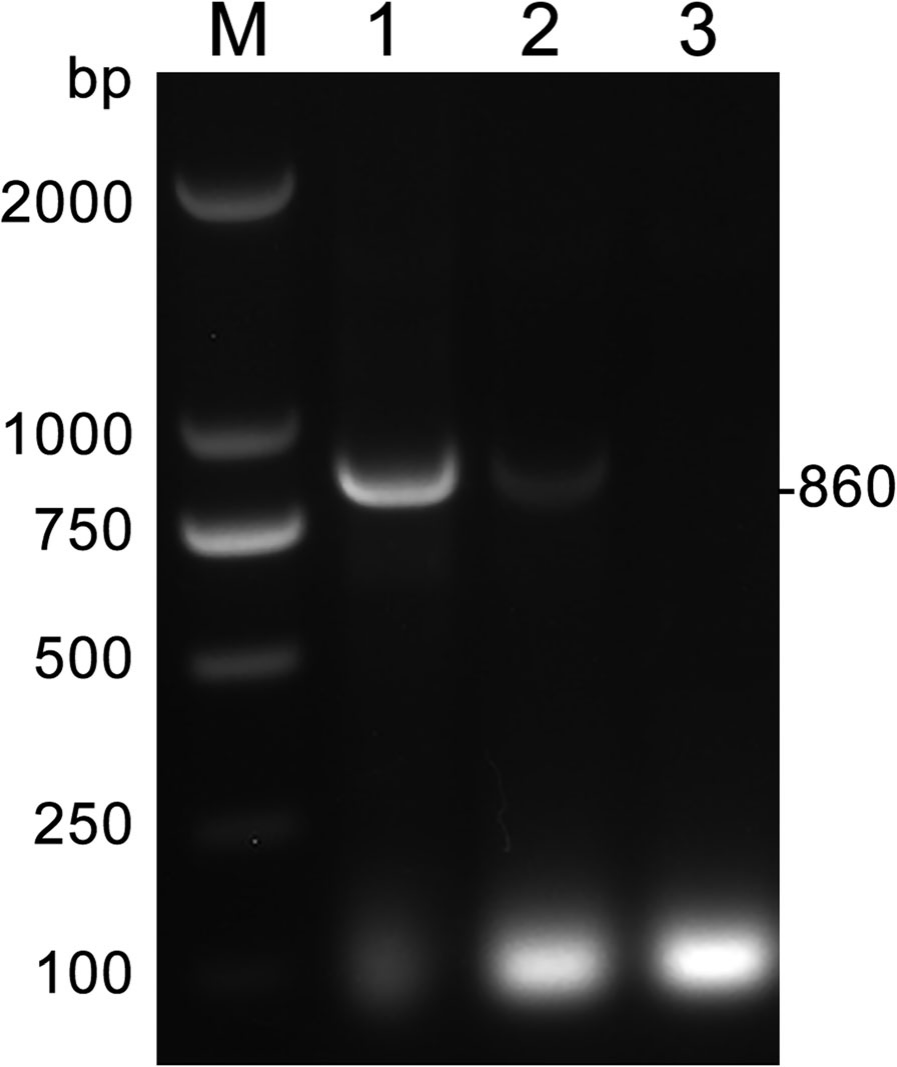
PCR products on *C. sinensis* adults and eggs. M, DNA marker; lane 1, *C. sinensis* adults; lane 2, *C. sinensis* eggs; lane 3, negative control. The arrow on the right side represents the amplicon of *C. sinensis*

A 52-year-old man who lived in Songyuan city, Jilin province, China, near the Songhua River and it is an endemic area of clonorchiasis. The man had an approximately twenty years history of eating raw freshwater fish during the annual summer fishing season but no history of other diseases, and he was hospitalized with acute shock caused by severe abdominal pain. After rescue, physical examination suggested the signs and symptoms of the patient included acute abdomen with severe abdominal pain in the right upper abdomen and rigidity, a drop in body temperature (35.2 °C), hyperhidrosis, vomiting, cyanosis of the mouth, increased respiratory rate (30–35 breaths per minute), increased heart rate (96–110 beats per minute), and decreased blood pressure (60–90/40–60 mmHg). Computed tomography (CT) revealed cholecystitis, and stone-like substances were observed in the gallbladder (Fig. 1), with no obvious symptoms in other abdominal organs. Magnetic resonance cholangiopancreatography (MRCP) revealed the same changes as seen in the CT results. Laboratory tests showed a leukocyte count of 7.03 × 10^9^/L (normal range, 3.5–9.5 × 10^9^/L) with an eosinophil percentage of 15.1 (normal range, 0.4–8.0%) and total bilirubin of 230.3 μmol/L (normal range, 6.8–30.0 μmol/L) with direct bilirubin of 116.9 μmol/L (normal range,0.0–8.6 μmol/L). Based on the clinical results and CT findings, we diagnosed the patient with a case of acute cholecystitis and cholelithiasis. Exploratory laparotomy and cholecystectomy were performed after admission. However, no stone-like substances were found. Instead, six liver flukes and parasite eggs were found in the bile. We found the morphology of the liver flukes to be consistent with *C. sinensis*, with an anterior oral sucker and a centrally located ventral sucker, dorso-ventrally flattened, slightly narrow front, blunt round rear, similar to a sunflower seed (Fig. 2). Eggs were similar to sesame seeds, light brown, with a thickening in the egg shell covering one side to form the shoulder (Fig. 2). Further, PCR was performed to identify the genomic DNA of the worms and eggs. Primers were designed based on the internal transcribed spacer 2 (ITS2) (GenBank No. JQ048577.1) of *C. sinensis* using Primer 5.0 software, and the fragment size was 860 bp (Fig. 3). Primer sequences are as follows: Primer F, TACCCAATATATATGATGTGC, and primer R, GAAAGTTAAGCACCGACCGGTGC. The sequencing results indicated that the DNA had 98% homology with *C. sinensis* China Heilongjiang isolates (GenBank no. KF740424.1). Therefore, the patient was diagnosed with clonorchiasis. The results of two blood cultures before and after the operation were negative. After emergency surgery, other examinations, including urine routine, urine culture, lung CT, stool routine, and CRP and PCT levels, were performed, and no other potential infectious lesions were found. The electrocardiogram was normal and did not support shock caused by myocardial infarction or cerebral infarction. Other possible causes of shock in this patient were ruled out. In addition, the prognosis was good after treating the patient with praziquantel for 30 days; no eggs were found in the stool, and the liver gradually restored normal function. Therefore, we concluded that *C. sinensis* infection may have been the primary cause of shock in this case.

## Discussion and conclusions

Here, we report a case of fatal shock caused by *C. sinensis* infection. *C. sinensis* seriously affects the health of people of East Asia, including those in China. National surveys on the status of serious human parasitic diseases in China demonstrated egg positive rates of 1.37–2.40% from 1990 to 2003 in the epidemic area [12]. It is predicted that 12.49 million people in mainland China have been infected with *C. sinensis*. The highly prevalent areas of clonorchiasis are found in the northeastern provinces of China, such as Liaoning, Jilin, Heilongjiang [1]. A survey in select townships of Jilin province demonstrated that fecal samples from approximately 8.90% of 910 people tested contained *C. sinensis* eggs [1]. Our recent epidemiological surveys indicated that the prevalence of egg-positive individuals was 29.40% of 1745 people by the Kato-Katz (KK) method in three townships near the Songhua River of Songyuan city, Jilin province (data unpublished). However, there is a paucity of comprehensive epidemiological information for Jilin province. For the past decade, several cases of clonorchiasis have been reported in Jilin province [13, 14]. Even with the high prevalence of clonorchiasis, few cases are diagnosed in clinical examination. Due to the non-specific and atypical symptoms and the fact that adult worms survive in the human body for up to 26 years, the disease has always been neglected and often leads to clonorchiasis [15, 16].

The patient in this study lived in Songyuan city, Jilin province, China, near the Songhua River, which is a heavily endemic area of clonorchiasis [17]. People living around the Songhua River have a deep-rooted custom of eating raw freshwater fish during festivals and feeding this food to cats and dogs, and the prevalence of *C. sinensis* is high. In the meantime, it is common in some endemic regions in China, particularly in the province of Jilin, for the “toilet” to be built close to fish ponds. Eggs of *C. sinensis* from human excrement are the source of infection for intermediate hosts in ponds [1]. The existence of a large number of intermediate and reservoir hosts is closely related to the maintenance and development of the complete life cycle of *C. sinensis* in local areas, leading to long periods of existence of the parasite [1]. Therefore, people around the Songhua River have a high prevalence of *C. sinensis* infection.

Chronic clonorchiasis in the gallbladder and bile ducts leads to various complications, such as cholecystitis, cholangitis, gallstones, and cholangiocarcinoma [18]. Several pathogenic mechanisms have been proven, such as the direct invasion of larva, injury caused by worms through adsorption or feeding activities on the mucosa of the bile ducts, and biliary obstruction by the worms and stones formed by excretory-secretory products (ESPs) or eggs [19–21]. Once the bile ducts are obstructed, this condition provides a favorable environment for secondary bacterial infection [6]. In addition, ESPs produced by worms increase free radicals, which could promote the production of pro-inflammatory cytokines through the NF-κB pathway [22]. Patients that presented with acute abdomen, calculous cholecystitis, jaundice and obstructive jaundice caused by *C. sinensis* infection have been reported in recent years [13, 14, 23]. However, acute shock caused by *C. sinensis* infection was not reported. In this rare case, acute cholecystitis caused by *C. sinensis* resulted in shock, which is a rare symptom of clonorchiasis. Not considering the life history of the patient in the epidemic area and neglecting the increase in eosinophil count led to misdiagnosis. Even if cholecystectomy is feasible, conservative antihelmintic treatment should be tried first.

The traditional diagnosis method of clonorchiasis is to detect the characteristic operculated ova in stool. This method is simple, rapid, and inexpensive; however, it is difficult to find eggs during early infection and biliary obstruction [24]. Patients with low-intensity and moderate-intensity infection had a higher rate of misdiagnosis, for which rich practical experience was needed. In China, it has been reported that the misdiagnosis rate of individuals with clonorchiasis was 57.1% of 49 patients, 52.5% of 40 patients, and 84.18% of 196 patients in different endemic areas [25–27]. Clonorchiasis was mainly misdiagnosed as hepatitis, cholecystitis, and gallstones [27]. Imaging and serological diagnoses are used as auxiliary methods to improve the diagnosis [28]. PCR is a complementary tool used for the diagnosis that can detect *C. sinensis* in patients with low-intensity infection and may help eliminate cross-reactions between the pathogen and other parasite species [29]. Therefore, we used PCR for diagnosis in this case.

In conclusion, shock caused by *C. sinensis* is rare in the clinical environment. This case improves our awareness of the impacts of clonorchiasis. The misdiagnosis rate of clonorchiasis is high in the clinical environment because the disease has no typical symptoms or is asymptomatic. Moreover, auxiliary methods are difficult to accurately diagnose without detecting eggs in the stool. Therefore, when a patient present with the above mentioned symptoms and imaging shows cholecystitis, cholelithiasis, increased bilirubin and eosinophils in blood, *C. sinensis* infection should be considered. Most importantly, this detection method will help reduce the misdiagnosis of clonorchiasis and unnecessary cholecystectomy.

## Data Availability

All data generated or analysed during this study are included in this published article.

## Abbreviations

CT: Computed Tomography
MRCP: Magnetic Resonance Cholangiopancreatography
PCR: Polymerase Chain Reaction
